# Latent profile analysis of nurses’ knowledge, attitudes, and practices regarding pressure injury prevention: a multicenter large-sample study

**DOI:** 10.1186/s12912-025-03875-3

**Published:** 2025-09-29

**Authors:** Caili Li, Xiaoqian Lu, Liyan Zhang, Xiuju Huang, Pinyue Tao, Xiao Pan, Hong Li, Tian Tian, Yanfei Pan, Qini Pan, Dongmei Huang, Dandan Han, Huiqiao Huang

**Affiliations:** 1 General Surgery, The Second Affiliated Hospital of Guangxi Medical University, Nanning, China; 2 Nursing Department, The Second Affiliated Hospital of Guangxi Medical University, Nanning, China; 3 Anesthesiology Department, The Second Affiliated Hospital of Guangxi Medical University, Nanning, China; 4 Ear, Nose, Throat, Head and Neck Surgery, The Second Affiliated Hospital of Guangxi Medical University, Nanning, China; 5 Cardiovascular Medicine Department, The Second Affiliated Hospital of Guangxi Medical University, Nanning, China; 6 Orthopedic Trauma Surgery, The Second Affiliated Hospital of Guangxi Medical University, Nanning, China; 7 Emergency Department, The Second Affiliated Hospital of Guangxi Medical University, Nanning, China; 8 Party Committee Office, The Second Affiliated Hospital of Guangxi Medical University, Nanning, China

**Keywords:** Pressure injury (PI), Nurses' knowledge, Attitudes and practices (KAP), Latent profile analysis, Influencing factors

## Abstract

**Background:**

This study aimed to analyze latent profiles and characteristics of nurses’ knowledge, attitudes, and practices (KAP) regarding pressure injury (PI) prevention, as well as influencing factors across distinct profiles.

**Methods:**

A convenience sampling method was employed to recruit nurses from hospitals at various tiers in Guangxi Zhuang Autonomous Region between July and August 2024. Data were collected using a General Information Questionnaire and a Nurse PI-KAP Questionnaire. Latent profile analysis (LPA) identified distinct PI-KAP profiles, while univariate analysis and multinomial logistic regression determined profile-specific influencing factors.

**Results:**

Among 17,253 enrolled nurses, the total PI-KAP score was 63.44 ± 7.69. Three latent profiles emerged: low-level PI-KAP (12.82%), moderate-level PI-KAP (52.23%), and high-level PI-KAP (34.95%). Multinomial logistic regression revealed that hospital tier, years of experience, education level, professional title, gender, and attitudes toward PI training significantly influenced PI-KAP profiles (*p* < .05).

**Conclusion:**

Heterogeneity exists in nurses’ PI-KAP profiles, with a substantial proportion demonstrating suboptimal competency. Nursing administrators should establish hierarchical training systems tailored to PI-KAP characteristics. Capacity-building strategies include prioritizing training for core nurses, optimizing resource allocation, and establishing tiered hospital assistance mechanisms to enhance team-based PI prevention capabilities.

**Clinical trial number:**

Not applicable.

**Supplementary Information:**

The online version contains supplementary material available at 10.1186/s12912-025-03875-3.

## Introduction

Pressure injury (PI), also termed pressure ulcer (PU), refers to localized damage to the skin and/or underlying tissues caused by prolonged pressure or a combination of pressure and shear forces [1]. PI represents a prevalent clinical issue in healthcare settings, contributing not only to patient suffering, psychological distress (e.g., pain and anxiety), but also to prolonged hospitalization, elevated healthcare costs, increased mortality risk, and systemic burden on medical institutions [2–4]. For instance, Australian public hospitals incurred an annual financial burden of approximately AU$9.11 billion in 2020 due to PI-related complications, of which AU$3.59 billion was allocated to direct treatment expenditures [3]. Similarly, in the United States, the annual cost attributed to PI management exceeds $17.8 billion, underscoring its substantial economic impact [2].

Globally, the prevalence of PI remains alarmingly high. A systematic review and meta-analysis involving 1,366,848 patients reported a pooled global PI prevalence of 12.8%, with an 8.4% incidence rate of hospital-acquired PI among 1,893,593 cases, reflecting substantial clinical burden [5]. Furthermore, studies from some countries document PI incidence rates of 14.9% in Sweden, 10.1% in São Paulo (Brazil), and 12.9% in Australia [3, 6, 7]. In China, the surveys indicate an overall PI prevalence of 12.26% among intensive care unit patients, with epidemiological trends demonstrating a progressive increase over time [8, 9]. These findings collectively underscore the pervasive global impact of PI on patient health outcomes and its persistent prioritization in medical research and clinical practice.

Nurses serve as the first line of defense in pressure injury (PI) prevention, playing a pivotal role in maintaining skin integrity and implementing effective prevention strategies [10]. As key developers, implementers, and supervisors of PI prevention protocols, nurses directly influence prevention outcomes and patient recovery quality. A Danish quality improvement initiative led by wound care nurse specialists successfully reduced PI prevalence from 17.3 to 2% over six years through hospital-wide implementation of comprehensive prevention measures [11]. Similarly, nurse-led prevention programs demonstrated significant efficacy, with hospital-acquired pressure injury (HAPI) rates decreasing from 1.177 to 0.272 per 1,000 patient-days [12]. The study by Öznur Erbay Dallı found that targeted training on medical device-related pressure injuries (MDRPIs) enhanced ICU nurses’ knowledge and preventive practices, subsequently reducing MDRPI incidence. The study by Öznur Erbay Dallı found that training on medical device-related pressure injuries (MDRPI) for ICU nurses improved their knowledge and preventive performance, which led to a reduction in the prevalence of MDRPIs [13].

These studies demonstrate the critical role of correct and effective nursing practices in preventing PI, with nurses’ knowledge and attitudes toward PI being key determinants of prevention and intervention adherence. Although numerous investigations have examined factors influencing nurses’ KAP regarding PI—providing pivotal evidence for enhancing care quality and mitigating complications [14–16]—the stratification of KAP levels remains underexplored. Previous studies have frequently analyzed the nursing population as a relatively homogeneous entity, primarily focusing on average KAP levels or unidimensional influencing factors. While this approach aids in understanding general trends and associations, it tends to overlook individual variations, potentially leading to substantial heterogeneity within the study findings.

Latent Profile Analysis (LPA) serves as a robust method for classifying participants into distinct subgroups based on specified variables [17]. This approach categorizes the study population through model fitting, thereby objectively revealing heterogeneity among individuals and facilitating the characterization of different subgroup profiles. This underscores the necessity of introducing LPA in the present study. Unlike traditional variable-centered approaches such as regression or mean comparison methods, LPA moves beyond focusing on aggregate averages to identify nurse subgroups with lower overall KAP levels. This capability provides a critical foundation for future tailored interventions, resource optimization, and management decision-making. Therefore, This study aimed to identify the latent profiles of nurses’ knowledge, attitudes, and practices regarding pressure injury prevention and to explore the key factors influencing these distinct profiles.

## Method

### Study design and participants

This cross-sectional study employed convenience sampling to recruit nurses from multi-level hospitals across Guangxi Zhuang Autonomous Region between July and August 2024. Inclusion criteria required participants to: (1) Possession of a nursing qualification certificateh, and (2) voluntary participation.

### Survey instruments

#### Demographic questionnaire

The survey form was self-designed by the researchers through literature review, including gender, age, years of work experience, professional title, highest level of education, hospital level, and training needs of PI.

#### PI prevention knowledge, attitudes, and practices questionnaire

The initial draft of the nurse questionnaire on prevention of pressure injuries was developed based on Hu’s [18] Nurse Knowledge, Attitude, and Practice Questionnaire for Preventing Medical Device-Related Pressure Injuries in Critically Ill Patients, adapted through literature review and aligned with this study’s objectives and content. Six clinical nursing experts and nursing management specialists participated in a panel discussion to finalize the questionnaire. The finalized instrument comprises three dimensions: knowledge (6 items), attitude (5 items), and practice (4 items), totaling 15 items. Each item employs a 5-point Likert scale (1–5 points), with total scores ranging from 15 to 75. Dimension-specific score ranges are: knowledge (6–30), attitude (5–25), and practice (4–20). Authorization for the use of the scale in this study was obtained from its original author.

The questionnaire exhibited strong reliability with a total Cronbach’s α coefficient of 0.935. The Cronbach’s α coefficients for the knowledge, attitude, and practice subscales were 0.940, 0.867, and 0.915, respectively. Exploratory factor analysis demonstrated a Kaiser-Meyer-Olkin (KMO) value of 0.929 and Bartlett’s test of sphericity (χ² = 225,339.61, *P* < .001), indicating suitability for factor analysis. Principal component analysis with varimax rotation extracted three common factors with eigenvalues > 1, collectively explaining 75.63% of the total variance. All items demonstrated factor loadings > 0.5.

Confirmatory factor analysis using maximum likelihood estimation was performed according to Wu’s [19] criteria: χ²/df < 5; root mean square error of approximation (RMSEA) < 0.08 (good fit), 0.08–1.00 (acceptable); root mean square residual (RMR) < 0.05; Tucker-Lewis index (TLI), comparative fit index (CFI), and incremental fit index (IFI) > 0.9, collectively suggesting satisfactory model fit. Initial model fit indices showed marginal acceptability (RMSEA = 0.096, RMR = 0.034, CFI = 0.94, TLI = 0.93, IFI = 0.94). The elevated χ²/df ratio (158.57) may reflect sensitivity to the large sample size [20]. To address this, two random subsamples were analyzed (N₁ = 400; N₂ = 500), yielding improved fit indices: For Sample 1: RMSEA = 0.089, RMR = 0.037, CFI = 0.95, TLI = 0.94, IFI = 0.95; for Sample 2: RMSEA = 0.088, RMR = 0.032, CFI = 0.95, TLI = 0.94, IFI = 0.95. Notably, the χ²/df ratios (4.13 and 4.84, respectively) decreased to acceptable thresholds.

### Data collection methods

Data were collected using Wenjuanxing (https://www.wjx.cn/), a professional online survey platform. The project coordinator coordinated communication with heads of nursing departments across participating hospitals. After explaining the study objectives, target population, and questionnaire administration procedures, formal approval was obtained from each hospital’s nursing department. Subsequently, the Wenjuanxing-generated questionnaire link and QR code were distributed to participants via WeChat, a Chinese instant messaging application.

The online questionnaire featured guidance instructions on the first page, detailing the research purpose, methodology, completion guidelines, and confidentiality principles. The second page contained a general information survey, followed by the Nurses’ Knowledge, Attitudes, and Practices Questionnaire on Pressure Injury Prevention on the third page. All questionnaire items were configured as mandatory fields. If participants attempted submission with incomplete responses, the system automatically prompted them to complete missing items. Technical controls restricted submission to one response per device and IP address. Researchers monitored the backend data in real-time and closed the survey after five consecutive days without new submissions. Electronic consent was implied through participants’ voluntary access of the survey link and questionnaire completion. Following data collection, dual researchers performed data verification and organization to ensure accuracy.

### Statistical analysis

Latent profile analysis (LPA) was performed using Mplus 8.3 to identify distinct subgroups of nurses’ knowledge, attitudes, and practices in pressure injury prevention. The analysis began with a one-class model, and the number of latent classes was incrementally increased. Model fit was evaluated by comparing the following indices: (1) Akaike information criterion (AIC), Bayesian information criterion (BIC), and adjusted Bayesian information criterion (aBIC), where smaller values indicate better model fit; (2) entropy, a measure of classification accuracy ranging from 0 to 1 (values > 0.8 indicate excellent precision); and (3) the Lo-Mendell-Rubin likelihood ratio test (LMRT) and bootstrapped likelihood ratio test (BLRT), where a statistically significant result (*P* < .05) suggests that the k-class model is superior to the (k − 1)-class model.

Confirmatory factor analysis (CFA) was conducted using SPSS AMOS version 24.0. Statistical analysis was performed using SPSS 23.0. Continuous data are presented as mean ± standard deviation. Intergroup comparisons were analyzed using one-way analysis of variance (ANOVA). For post-hoc pairwise comparisons, the LSD test was used when the assumption of homogeneity of variances was met; otherwise, Tamhane’s T2 test was applied. Categorical variables were summarized as frequencies (%) and analyzed using chi-square tests or Wilcoxon rank-sum tests, as appropriate. Multivariate logistic regression was employed to identify factors influencing the distinct KAP profiles identified in the LPA. The statistical significance level was set at α = 0.05.

## Results

### General characteristics of participants

As of August 31, 2024, a total of 17,613 nurses were invited to participate in this study. Among these, 17,253 valid responses were collected, yielding an effective rate of 97.96%. The general characteristics of the participants are summarized in Table 1.

**Table 1:** General characteristics of participants and univariate analysis of latent profiles in pressure injury prevention knowledge, attitudes, and practices (*N* = 17,235)

Category	*N* (%)	Low-level PI-KAP *N* (%)	Moderate-level PI-KAP *N* (%)	High-level PI-KAP *N* (%)	Test Statistic	*p*
Age						
18–30 years	8504 (49.34)	1007 (45.57)	4546 (50.51)	2951 (48.99)	32.403	<.001^a^
30–40 years	6931 (40.21)	904 (40.90)	3643 (40.47)	2384 (39.58)		
40–50 years	1417 (8.22)	226 (10.23)	659 (7.32)	532 (8.83)		
>50 years	383 (2.22)	73 (3.30)	153 (1.70)	157 (2.61)		
Gender						
Male	615 (3.57)	68 (3.08)	282 (3.13)	265 (4.40)	18.590	<.001^b^
Female	16,620 (96.43)	2142 (96.92)	8719 (96.87)	5759 (95.60)		
**Years of work experience**						
≤ 1 year	1169 (6.78)	170 (7.69)	631 (7.01)	368 (6.11)	28.704	<0.001 ^a^
> 1 to ≤ 10 years	9379 (54.42)	1116 (50.50)	5028 (55.86)	3235 (53.70)		
> 10 to ≤ 20 years	5237 (30.39)	694 (31.40)	2686 (29.84)	1857 (30.83)		
> 20 to ≤ 30 years	1042 (6.05)	155 (7.01)	495 (5.50)	392 (30.83)		
> 30 years	408 (2.37)	75 (3.39)	161 (1.79)	172 (2.86)		
**Professional title**						
Nurse	4059 (23.55)	466 (21.09)	2101 (23.34)	1492 (24.77)	18.933	<0.001 ^a^
Senior nurse	6703 (38.89)	835 (37.78)	3516 (39.06)	2352 (39.04)		
Supervisor nurse	5668 (32.89)	799 (36.15)	2981 (33.12)	1888 (31.34)		
Co-chief nurse or above	805 (4.67)	110 (4.98)	403 (4.48)	292 (4.85)		
**Highest level of education**						
Technical secondary school	253 (1.47)	49 (2.22)	100 (1.11)	104 (1.73)	30.363	<0.001 ^a^
Junior college	4851 (28.15)	636 (28.78)	2408 (26.75)	1807 (30.00)		
Bachelor’s Degree or above	12,131 (70.39)	1525 (69.00)	6493 (72.14)	4113 (68.28)		
**Hospital level**						
Grade IIB hospital or below	1084 (6.29)	188 (8.51)	512 (5.69)	384 (6.37)	108.459	<0.001 ^a^
Grade IIA hospital	7578 (43.97)	1121 (50.72)	4039 (44.87)	2418 (40.14)		
Grade IIIB hospita	1295 (7.51)	152 (6.88)	698 (7.75)	445 (7.39)		
Grade IIIA hospital	7278 (42.23)	749 (33.89)	3752 (41.68)	2777 (46.10)		
**Training needs of PI**						
Yes	15,604 (90.54)	1968 (89.05)	8023 (89.13)	5613 (93.18)	75.383	<0.001 ^b^
No	1631 (9.46)	242 (10.95)	978 (10.87)	411 (6.82)		

Note: Rank sum test for a; X^2^ test for b

### Scores of knowledge, attitudes, and practices in pressure injury prevention

Significant differences were observed in PI knowledge, attitude, and practice dimension scores, as well as total scores, among the three distinct PI-KAP level groups (*p* < .001). Post-hoc pairwise comparisons revealed that the high-level PI-KAP group demonstrated significantly higher scores across all dimensions and total scores compared to both the medium-level and low-level PI-KAP groups (*p* < .001). Furthermore, the medium-level PI-KAP group exhibited significantly higher dimension scores and total scores than the low-level PI-KAP group (*p* < .001). Detailed results are presented in Table 2.

**Table 2:** Scores of nurses’ knowledge, attitudes, and practices in pressure injury prevention (*N* = 17,235)

Variable	Total	Low-level PI-KAP (M ± SD)	Moderate-level PI-KAP (M ± SD)	High-level PI-KAP (M ± SD)	Test Statistic	*p*
Knowledge Dimension	24.10 ± 4.09	19.56 ± 3.56	23.21 ± 3.13^#&^	27.09 ± 3.30^#^	4638.223	<0.001^*^
Attitude Dimension	22.65 ± 2.35	20.61 ± 2.69	22.05 ± 2.10^#&^	24.31 ± 1.33^#^	4363.629	<0.001^*^
Practice Dimension	16.70 ± 2.64	11.92 ± 1.50	15.94 ± 0.87_#&_	19.58 ± 0.67^#^	58122.26	<0.001^*^
Total KAP Score	63.44 ± 7.69	52.09 ± 5.57	61.20 ± 4.40^#&^	71.00 ± 4.17^#^	15256.594	<0.001^*^

Note: (1)* When variances were heterogeneous, Welch’s correction was applied

(2) Tamhane’s T2 test: # *p* < .001 vs. Low-level PI-KAP; &*p* < .001 vs. High-level PI-KAP

### Results of latent profile analysis for pressure injury prevention KAP

In this study, models with 1 to 4 latent classes were fitted using the three KAP dimensions as manifest indicators. The fit indices for each model are presented in Table 3. Starting with a single-class model, the AIC, BIC, and aBIC values progressively decreased as the number of classes increased. When the number of classes reached 4, the LMRT was statistically nonsignificant (*P* > .05). For the 2-class model, the entropy value (0.81) was markedly lower than that of the 3-class model (0.93). The 3-class model demonstrated smaller AIC, BIC, and aBIC values, higher entropy, and statistically significant LMRT and BLRT results (*P* < .001). Additionally, the average latent class probabilities for the 3-class model were 95.20%, 96.40%, and 97.60%, indicating high classification reliability. Collectively, the three-class model was identified as the optimal solution.

**Fig. 1:**
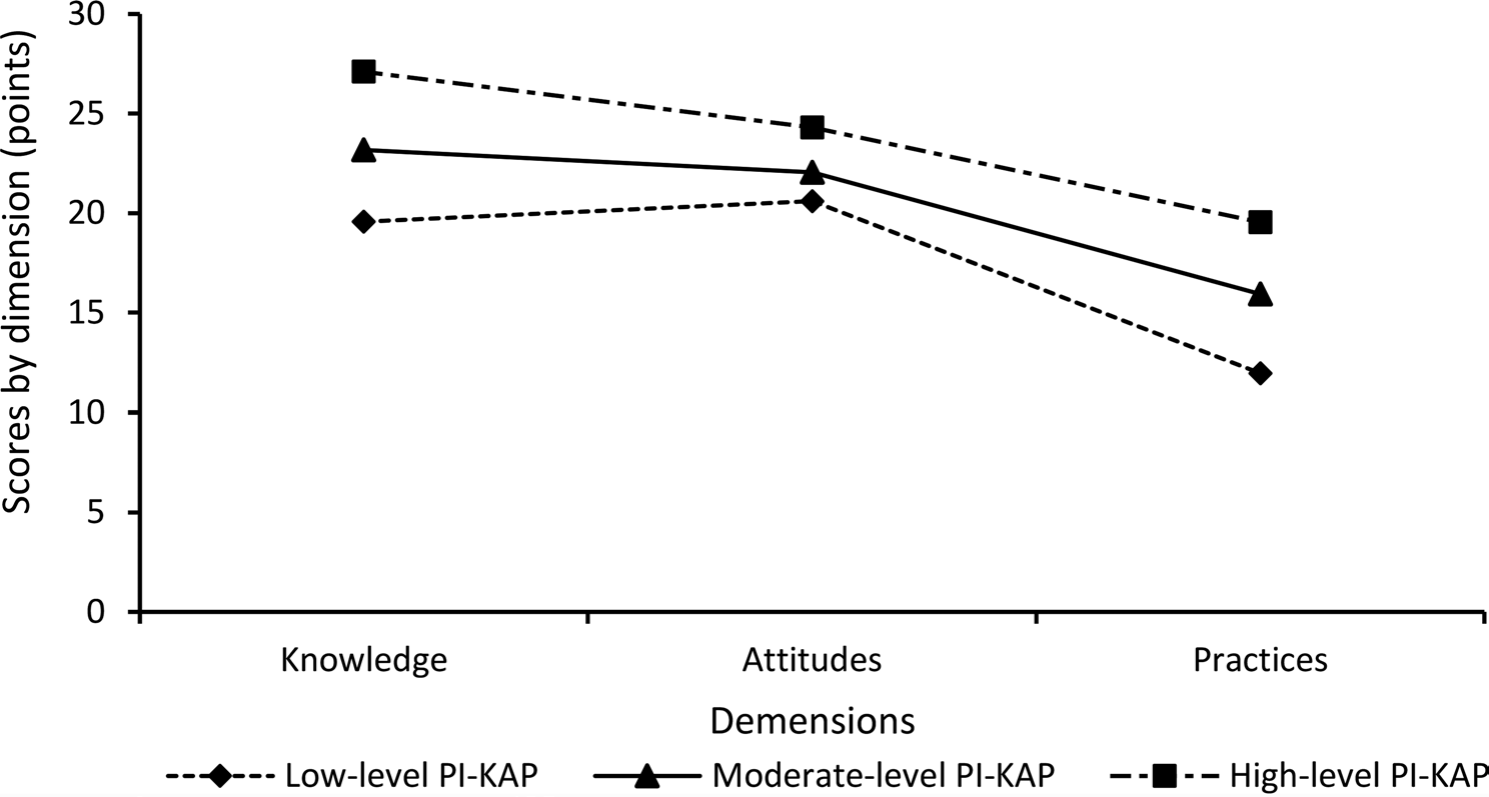
Distribution characteristics of latent profiles in nurses’ KAP on pressure injury prevention

The mean scores of the three latent profiles across the KAP dimensions are illustrated in Fig. 1. Based on their distinct characteristics, the profiles were labeled as follows:

Profile 1 (Low-level PI-KAP): Participants in this profile (*n* = 2,210, 12.82%) exhibited the lowest scores across all three KAP dimensions.

Profile 2 (Moderate-level KAP): Participants in this profile (*n* = 9,001, 52.23%) demonstrated intermediate scores across all dimensions.

Profile 3 (High-level PI-KAP): Participants in this profile (*n* = 6,024, 34.95%) achieved the highest scores in all dimensions.

**Table 3:** Fit indices of the latent profile model for nurses’ KAP in pressure injury prevention (*N* = 17,235)

Model (Number of Classes)	AIC	BIC	aBIC	LMRT *p*-value	BLRT *p*-value	Entropy	Class proportions (%)
1	258269.867	258316.395	258297.328	N/A	N/A	N/A	N/A
2	245177.727	245255.274	245223.494	<0.001	<0.001	0.81	46.70%/53.30%
3	239972.868	240081.434	240036.943	<0.001	<0.001	0.925	12.82%/52.23%/34.95%
4	236033.717	236173.301	236116.099	0.08	<0.001	0.95	29.86%/2.95%/45.76%/21.43%

Note: Entropy values range from 0 to 1. An entropy ≥ 0.8 indicates approximately 90% classification accuracy, with values approaching 1 signifying higher classification precision

### Multivariate analysis of latent profiles in nurses’ pressure injury prevention KAP

#### Multicollinearity assessment

To evaluate the suitability of selected explanatory and control variables for regression analysis, variance inflation factors (VIF) were computed to quantify multicollinearity. Consistent with established methodological standards, VIF values between 0 and 5 indicate acceptable collinearity levels. In this study, VIF statistics ranged from 1.004 to 3.436 (Table 4), confirming the absence of substantial multicollinearity among independent variables.

**Table 4:** VIF analysis

Variables	VIF	1/VIF
Gender	1.03	0.971
Age	3.373	0.296
Years of work experience	3.436	0.291
Highest level of education	1.222	0.818
Professional title	2.308	0.433
Hospital level	1.037	0.964
Training needs of PI	1.004	0.996

#### Multivariate analysis of latent profiles in nurses’PI- KAP

Variables with statistical significance in the univariate analysis were included as independent variables in a multivariate logistic regression model (Table 5). The coding scheme for variables was as follows: (1)Age:18 < Age ≤ 30 = 1, 30 < Age ≤ 40 = 2, 40 < Age ≤ 50 = 3, Age > 50 = 4; (2)Gender: Male = 1, Female = 2; (3)Years of work experience: ≤1 year = 1, > 1 to ≤ 10 years = 2, > 10 to ≤ 20 years = 3, > 20 to ≤ 30 years = 4, > 30 years = 5; (4)Professional title: nurse = 1, senior nurse = 2, supervisor nurse = 3, co-chief nurse or above = 4; (5)Highest level of education: technical secondary school = 1, Junior college = 2, Bachelor’s Degree or above = 3; (6)Hospital level: Grade IIB hospital or below = 1, Grade IIA hospital = 2, Grade IIIB hospital = 3, Grade IIIA hospital = 4; (7)Training needs of PI: Yes = 1, No = 2. In SPSS, the highest coded value was set as the reference category by default.

**Table 5:** Logistic regression analysis of three latent profiles in nurses’ knowledge, attitudes, and practices (KAP) on pressure injury prevention (*N* = 17,235)

Variable	B	SE	Wald	OR	95% CI		*p*
					Lower Bound	Upper Bound	
**Class3 vs. Class1** ^**I**^							
Intercept	-0.608	0.189	10.395				0.001
Gender = Male	-0.301	0.142	4.471	0.74	0.56	0.978	0.034
Years of work experience ≤ 1 year	1.14	0.342	11.15	3.128	1.602	6.109	0.001
Professional title = nurse	-0.408	0.168	5.871	0.665	0.478	0.925	0.015
Hospital level= Grade IIB hospital or below	0.683	0.1	46.641	1.98	1.627	2.408	<0.001
Hospital level= Grade IIA hospital	0.591	0.056	111.474	1.806	1.618	2.015	<0.001
Hospital level= Grade IIIB hospita	0.295	0.103	8.135	1.343	1.097	1.645	0.004
Training needs of PI = Yes	-0.584	0.086	46.024	0.558	0.471	0.66	<0.001
**Class2 vs. Class1** ^**II**^							
Intercept	-1.122	0.183	37.483				<0.001
18 < Age ≤ 30 = 1	-0.712	0.32	4.964	0.49	0.262	0.918	0.026
technical secondary school	0.456	0.189	5.831	1.578	1.09	2.286	0.016
Hospital level= Grade IIB hospital or below	0.598	0.096	38.855	1.818	1.507	2.194	<0.001
Hospital level= Grade IIA hospital	0.327	0.053	37.545	1.387	1.249	1.54	<0.001

Note: (1)Class1: Low-level PI-KAP; Class2: Moderate-level PI-KAP; Class2: High-level PI-KAP

(2) Model I: Class 3 served as the reference category; Model II以Class2 served as the reference category

## Discussion

This research aimed at analyzing the differences in the levels of KAP regarding pressure injuries among nurses based on latent profiles. The results indicated that, using LPA techniques, three distinct latent profile groups were identified based on nurses’ PI-KAP scores: the Low-level PI-KAP group, Moderate-level PI-KAP group, and High-level PI-KAP group. Logistic regression analysis revealed that Hospital level, Years of work experience, Highest level of education, Professional title, Gender, and Training needs of PI were significant factors influencing nurses’ PI-KAP levels. Specifically, nurses with Years of work experience ≤ 1 year (OR: 3.128; 95%CI: 1.602–6.109), those working in Grade IIB hospitals or below (OR: 1.98; 95%CI: 1.627–2.408), Grade IIA hospitals (OR: 1.806; 95%CI: 1.618–2.015), or Grade IIIB hospitals (OR: 1.343; 95%CI: 1.097–1.645) (compared to the High-level PI-KAP group as the reference), and those with a Highest level of education of technical secondary school (OR: 1.579; 95%CI: 1.09–2.286) or employed in Grade IIB hospitals or below (OR: 1.818; 95%CI: 1.507–2.194) or Grade IIA hospitals (OR: 1.387; 95%CI: 1.249–1.54) (compared to the Moderate-level PI-KAP group as the reference) had significantly higher odds of belonging to the low PI-KAP group. Thus, the identification of nurses with varying PI-KAP levels and their defining characteristics provides a reference for nursing administrators to develop targeted intervention strategies and management decisions to enhance PI prevention capabilities.

The Low-level PI-KAP group constituted 12.82% of the sample (*n* = 17,235), with the lowest total PI-KAP score (52.09 ± 5.57). This profile exhibited significantly lower scores across all dimensions compared to other groups, revealing critical gaps in pressure injury-related cognition and clinical practice among these nurses. In contrast, the Moderate-level PI-KAP group constituted the majority of the sample (52.23%, *n* = 17,235), achieving a mean total score of 61.20 ± 4.40. This predominance suggests that over half of nurses in Guangxi Zhuang Autonomous Region exhibit intermediate-level PI-KAP competencies. The High-level PI-KAP group accounted for 34.95% (*n* = 17,235) of participants, attaining the highest overall score (71.00 ± 4.17). Distinct from the first profile, these nurses outperformed counterparts in all three domains, reflecting their solid theoretical knowledge, positive attitudes, and efficient execution capabilities in PI prevention. Therefore, it can be concluded that the majority of nurses in Guangxi Zhuang Autonomous Region currently demonstrate moderate to low levels of PI-related KAP, indicating substantial potential for enhancement.

Furthermore, within the latent profile distribution characteristics map for PI prevention KAP (Fig. 1), we observed an interesting phenomenon: the three nurse subgroups exhibited concurrent graded patterns across all three KAP dimensions. Pronounced stratification differences were noted in the knowledge and practice dimensions, whereas the attitude dimension demonstrated minimal stratification. Similar patterns have been observed in other studies. A Croatian investigation reported nurses’ correct knowledge rate regarding PI was below 50% [21]. Another study from Indonesia revealed that while nurses exhibited a satisfactory attitude toward PI (75.46%), their correct knowledge rate stood at merely 35.02% [22]. Furthermore, a KAP survey of pediatric intensive care unit nurses on PI prevention demonstrated positive attitudes toward prevention, yet insufficient knowledge and practice [23]. The Knowledge-Attitude-Practice (KAP) framework, a widely utilized theoretical model in health behavior research, conceptualizes behavioral change as a sequential process encompassing knowledge acquisition, belief formation, and behavior adoption [24]. This posits knowledge as the foundation, belief as the impetus, and behavioral change as the ultimate objective. Consequently, PI knowledge—serving as the cognitive basis and precursor to practice—directly influences nurses’ capacity to implement PI prevention. The minimal stratification differences observed in the attitude dimension, acting as the bridge between knowledge and practice, suggest that nurses may have developed a pervasive and stable internalized cognition regarding PI prevention importance, likely reinforced through professional norms. This further indicates that positive attitudes alone are insufficient to drive high-level practice. The acquisition, updating of PI knowledge, and its effective translation into practice remain critical components for enhancing nurses’ PI prevention capabilities.

Our study further revealed significant sociodemographic disparities across PI-KAP proficiency levels. Logistic regression analysis demonstrated that compared to the High-level PI-KAP group, nurses with ≤ 1 year of work experience (OR: 3.128; 95%CI: 1.602–6.109) and those employed in Grade IIB or below (OR: 1.98; 95%CI: 1.627–2.408), Grade IIA (OR: 1.806; 95%CI: 1.618–2.015), and Grade III-B hospitals (OR: 1.343; 95%CI: 1.097–1.645) exhibited significantly higher probabilities of belonging to the Low-level PI-KAP group. Notably, nurses within their first year of practice—typically in the novice stage—face intensive demands to acquire fundamental clinical skills (e.g., venipuncture, emergency procedures), which may lead to the deprioritization of PI prevention training as a “non-urgent competency.” This phenomenon aligns with findings by Li [25], whose study identified significant deficiencies in pressure injury care behaviors among early-career nurses.

In China, Grade III-A hospitals frequently assume responsibilities for advanced medical services, academic instruction, and clinical training. This strategic positioning enables greater access to premium medical-educational resources and enhances their capacity for pressure injury (PI) prevention through comprehensive toolkits. Empirical evidence consistently demonstrates that nurses in these Grade III-A hospitals exhibit superior PI prevention competencies compared to their counterparts in lower-tier facilities [14, 25, 26]. In contrast, nurses in Grade IIB and lower-tier hospitals, Grade IIA hospitals, and Grade IIIB hospitals are more likely to exhibit lower proficiency in PI-KAP.

Notably, male nurses (OR: 0.74;95%CI: 0.56–0.978), those holding nurse-level titles (OR: 0.665; 95%CI: 0.478–0.925), and nurses who recognize the need for PI training (OR: 0.558; 95%CI: 0.471–0.66) are more likely to exhibit high levels of PI-KAP. Compared to developed countries, the proportion of male nurses in China remains significantly below the international average. Male nurses in China have better career prospects and opportunities for promotion compared to female nurses [27]. This suggests that male nurses may receive more attention and are more likely to bear greater responsibilities and challenges, which could be a reason for the tendency of male nurses to have higher levels of PI-KAP. These findings align with Dirgar’s observations that male nurses demonstrate superior PI prevention knowledge assessment scores compared to female nurses [28]. However, this conclusion lacks consistent validation across studies. Notably, Lyu conversely found that female ICU nurses exhibited higher-level knowledge regarding MDRPI prevention [26]. While the precise etiology of this discrepancy remains unclear, one potential contributing factor may relate to the concentration of male nurses in departments with heightened physical, mental, and endurance demands (e.g., operating rooms, emergency departments, ICUs). A national survey on male nurses’ practice status in China revealed that among 10,676 male nurses, 6,646 (62.3%) were concentrated in just three departments: ICUs, emergency departments, and operating rooms [29]. These high-risk PI units typically manage patients with complex conditions, prolonged immobilization, and frequent medical device use. Such high-intensity clinical exposure environments may facilitate accelerated accumulation of practical experience and deepen understanding of PI prevention importance, consequently enabling male nurses to demonstrate comparatively higher levels of PI-KAP proficiency.

Nursing staff with primary-level titles displayed higher PI-KAP compared to those with associate senior titles or higher, a pattern consistent with findings from South Ethiopia [30]. However, other research has reported the opposite conclusion, indicating that nurses with higher titles demonstrate better PI knowledge and practice due to their richer clinical experience and greater exposure to PI cases, which fosters the accumulation of relevant nursing insights [31, 32]. It is noteworthy that nurses with associate senior or higher titles are typically older. Consequently, their frontline clinical work capacity may be relatively weaker [30], and their PI-KAP levels might be more influenced by past experience. Furthermore, they often bear heavier family responsibilities, potentially leading to diminished energy and motivation for learning, as well as a relatively weaker ability to assimilate new knowledge. The phenomenon observed in this study may stem from the following reasons: First, the majority of nursing staff at this stage likely have 1–5 years of work experience. Being younger, they possess greater adaptability, are less influenced by accumulated work experience, and are better able to apply theoretical knowledge to practice. Second, it may be related to the small proportion of nurses with associate senior or higher titles within the study sample.

Furthermore, nurses who perceived a need for PI training demonstrated higher levels of PI-KAP. This indicates a more positive attitude toward PI and an ability to objectively identify gaps in their own PI prevention practices. Concurrently, as medical knowledge continuously evolves and PI prevention/management guidelines are regularly updated, nurses require continuing education and training to maintain the currency of their professional knowledge. Given that nurses’ PI knowledge levels and attitudes are critical factors influencing their clinical decision-making related to prevention [33], this objective awareness of their knowledge and skill gaps serves as intrinsic motivation for learning and represents a possible explanation for their higher PI-KAP levelsCompared to moderate PI-KAP levels, nurses with final education credentials from technical secondary schools and those working in Grade IIB or below, Grade IIA hospitals demonstrated significantly higher probabilities of being classified as low-level PI-KAP. Jiang [34] found that nurses with advanced education (bachelor’s degree or higher) were 1.6 times more likely to possess adequate PI-prevention knowledge compared to those with diplomas or lower qualifications. Our findings corroborate this pattern, revealing that nurses with technical secondary education (lower diploma) exhibited inferior PI-KAP performance, suggesting educational background substantially influences nurses’ PI-related knowledge, attitudes, and practical competencies.

However, multiple studies [35–37] consistently demonstrate that enhanced PI-focused education and training interventions can improve nurses’ knowledge, modify clinical behaviors, and reduce PI incidence rates. Therefore, targeted continuing education and specialized training programs should be prioritized for nurses with lower educational attainment to elevate their PI-KAP proficiency, ultimately enhancing patient care quality and healthcare system outcomes.Compared to nurses over 50 years old, those aged 18–30 years showed increased likelihood of moderate PI-KAP classification. Empirical evidence indicates nursing staff above 40 years old exhibit 45% reduced probability of implementing optimal PI prevention practices compared to younger cohorts [30]. This pattern underscores the dual challenges of career burnout and outdated knowledge retention confronting mid-career nurses, necessitating focused institutional attention.

Our study revealed that nurses’ PI-KAP levels correlate with their work environment (hospital grade), professional development stage (years of experience, professional title), educational background (academic qualification), and learning motivation (attitude towards training). Consequently, understanding these differences enables nursing managers to precisely identify key cohorts influencing PI prevention competency, thereby providing a reference for developing tiered, differentiated, and targeted intervention strategies, resource allocation plans, and management systems. Nurses with low PI-KAP levels were characterized by lower final academic qualifications, limited work experience (particularly those with less than one year of service), and employment in non-tertiary Grade A medical institutions; this profile indicates a relatively weak foundational PI knowledge base, attitudes requiring reinforcement, and practices needing internalization within this group, identifying them as a critical vulnerability and priority target for enhancing PI prevention capacity. These findings offer implications for nursing management: for newly recruited nurses and those with lower academic qualifications, a tiered training system should be established for focused education, facilitated by developing a streamlined yet highly practical core PI prevention toolkit concentrating on fundamental and critical elements to enable rapid mastery and application; concurrently, regional healthcare consortiums should implement targeted support programs involving regular PI training courses to elevate prevention and control capabilities in non-tertiary Grade A hospital nursing teams, while broadening their learning channels, increasing resource accessibility, and promoting the decentralization of educational resources to grassroots levels. For highly motivated young nurses demonstrating high PI-KAP levels, emphasis should be placed on cultivating them as core personnel in PI prevention and management to mentor nurses with medium or low proficiency, supported through participation in high-level domestic and international academic conferences and specialized nurse training programs to maintain knowledge currency, thereby driving the overall advancement of PI skills across the nursing workforce.

To enhance PI prevention among nurses in Guangxi, we are undertaking several initiatives. Currently, we are developing a quality control toolkit specifically for the prevention of new stage 2 + pressure injuries in hospitalized patients within the Guangxi region. This toolkit encompasses eight core nursing interventions: assessment, skin management, positioning management, pressure redistribution device utilization, management of medical device-related pressure injuries, nutritional management, key points for shift handover, and nursing documentation. It aims to provide clinical nurses with highly referential and actionable clinical practice guidelines for PI prevention. Through the implementation of this toolkit, we anticipate enhancing nursing staff’s awareness of PI prevention and their professional skills, thereby driving improvements in nursing quality. Following the formal confirmation and release of the toolkit, we plan to promote and implement it through multi-level and multi-format channels, such as training workshops and academic conferences. Representative healthcare institutions will be selected for pilot application and effectiveness evaluation to verify the toolkit’s efficacy and feasibility. This will provide a basis for its standardized application and continuous improvement across Guangxi in subsequent phases. Furthermore, future work should explore personalized intervention models based on nurses’ KAP characteristics. By tracking outcome metrics such as patient pressure ulcer incidence rates and nurse adherence rates to preventive measures, the cost-effectiveness of different training strategies can be validated. This will ultimately facilitate the development of a scientifically grounded, tiered intervention pathway.

This study has several limitations. Firstly, the investigation exclusively collected PI-KAP data from nurses in hospitals across Guangxi Zhuang Autonomous Region, lacking representation from other provincial-level administrative units. Given China’s vast geographical span with substantial regional disparities in economic development levels, healthcare resource allocation, and nursing education systems, the generalizability of findings to economically advanced eastern regions or underdeveloped western provinces requires further verification. Secondly, as a cross-sectional survey employing self-reported measures, the data may incorporate response biases that deviate from actual clinical practices. Finally, the substantial sample size in this study increases statistical sensitivity, potentially detecting associations that are statistically significant yet of limited clinical relevance.

## Conclusion

This study established three latent profiles of nurses’ PI-KAP and identified their influencing factors. Our results indicate that the majority of nurses demonstrate moderate-to-low PI-KAP levels. Hospital level, years of work experience, highest level of education, professional title, gender, and training needs of PI were significant influencing factors of nurses’ PI-KAP levels. Nursing administrators should maintain targeted focus on nurses’ work environments, professional development stages, educational backgrounds, and learning motivation to identify the low-PI-KAP cohort. Implementation of stratified and differentiated precision support and intervention strategies—tailored to the characteristic distributions of distinct latent profiles—is essential, with priority given to enhancing PI prevention capabilities among low-proficiency groups. This approach will ultimately advance the holistic pressure injury prevention capacity across the nursing workforce.

## Supplementary Information

Below is the link to the electronic supplementary material.

Supplementary Material 1

## Data Availability

No datasets were generated or analysed during the current study.
