# Supplementary material for: Latent profile analysis of nurses’ knowledge, attitudes, and practices regarding pressure injury prevention: a multicenter large-sample study
Source: BMC Nurs. 2025 Sep 29;24:1213. doi: 10.1186/s12912-025-03875-3 (PMC12482050; doi:10.1186/s12912-025-03875-3)
Supplement: Supplementary file 1 — Supplementary Material 1 [file 12912_2025_3875_MOESM1_ESM.pdf]

PI Prevention Knowledge, Attitudes, and Practices Questionnaire

Dimensions	Items	Very familiar （5points）	Somewhat Familiar （4points）	Moderately Familiar （3points）	Have Heard Of （2points）	Not Familiar At All（1point）
Knowledge	Your level of understanding of the definition of pressure injuries.					
	Your level of understanding of the etiology of pressure injuries.					
	Your level of understanding of the common anatomical sites of pressure injuries.					
	Your level of understanding of the staging criteria for pressure injuries.					
	Your level of understanding of preventive measures for pressure injuries.					
	Your level of understanding of current advancements in pressure injury knowledge.					
		Very much agree （5points）	Agree （4points）	Neither agree nor disagree （3points）	Disagree （2points）	Strongly disagree （1point）
Attitude	You believe that attending training helps nurses better prevent the occurrence of pressure injuries.					
	You believe that training on pressure injury knowledge is very important.					
	You believe that attention should be paid to the prevention of pressure injuries in daily nursing care.					
	You believe that observation of patients with pressure injuries should be clearly communicated during every shift handover.					

	You believe that cases of pressure injuries should be reported in a timely manner.					
		Always able to do so （5points）	Often able to do so （4points）	Sometimes able to do so（3points）	Rarely able to do so （2points）	Never able to do so （1point）
Practice	You are able to monitor the clinical progression of pressure injuries.					
	You are capable of developing an appropriate management plan for patients with pressure injuries (e.g., regular repositioning, pressure-relieving devices, postural changes).					
	You can formulate correct treatment measures for pressure injuries through ongoing assessment.					
	Based on your current knowledge, you are able to make sound decisions regarding the prevention, assessment, and treatment of pressure injuries.					
